# Fibrin glue in the treatment of anal fistula: a systematic review

**DOI:** 10.1186/1750-1164-3-12

**Published:** 2009-11-14

**Authors:** Roberto Cirocchi, Eriberto Farinella, Francesco La Mura, Lorenzo Cattorini, Barbara Rossetti, Diego Milani, Patrizia Ricci, Piero Covarelli, Marco Coccetta, Giuseppe Noya, Francesco Sciannameo

**Affiliations:** 1 Department of General Surgery, St Maria Hospital, Terni, University of Perugia, Perugia, Italy; 2 Department of General and Emergency Surgery, St Maria Hospital, Perugia, University of Perugia, Perugia, Italy; 3 Department of General and Oncology Surgery, St Maria Hospital, Perugia, University of Perugia, Perugia, Italy

## Abstract

**Background:**

New sphincter-saving approaches have been applied in the treatment of perianal fistula in order to avoid the risk of fecal incontinence. Among them, the fibrin glue technique is popular because of its simplicity and repeatability. The aim of this review is to compare the fibrin glue application to surgery alone, considering the healing and complication rates.

**Methods:**

We performed a systematic review searching for published randomized and controlled clinical trials without any language restriction by using electronic databases. All these studies were assessed as to whether they compared conventional surgical treatment versus fibrin glue treatment in patients with anal fistulas, in order to establish both the efficacy and safety of each treatment. We used Review Manager 5 to conduct the review.

**Results:**

The healing rate is higher in those patients who underwent the conventional surgical treatment (P = 0,68), although the treatment with fibrin glue gives no evidence of anal incontinence (P = 0,08). Furthermore two subgroup analyses were performed: fibrin glue in combination with intra-adhesive antibiotics versus fibrin glue alone and anal fistula plug versus fibrin glue. In the first subgroup there were not differences in healing (P = 0,65). Whereas in the second subgroup analysis the healing rate is statistically significant for the patients who underwent the anal fistula plug treatment instead of the fibrin glue treatment (P = 0,02).

**Conclusion:**

In literature there are only two randomized controlled trials comparing the conventional surgical management versus the fibrin glue treatment in patients with anal fistulas. Although from our statistical analysis we cannot find any statistically significant result, the healing rate remains higher in patients who underwent the conventional surgical treatment (P = 0,68), and the anal incontinence rate is very low in the fibrin glue treatment group (P = 0,08). Anyway the limited collected data do not support the use of fibrin glue. Moreover, in our subgroup analysis the use of fibrin glue in combination with intra-adhesive antibiotics does not improve the healing rate (P = 0.65), whereas the anal fistula plug treatment compared to the fibrin glue treatment shows good results (P = 0,02), although the poor number of patients treated does not lead to any statistically evident conclusion. This systematic review underlines the need of new RCTs upon this issue.

## Background

The anal fistula, as a chronic inflammatory process, does not heal spontaneously. Although the conservative management, which consists of antibiotic therapy against the Gram-negative organisms and anaerobic bacteria, may be effective in the acute and early phase of the anal disease, surgery remains the elective treatment. Since 400 BC, when Hippocrates described a fistulotomy and the employment of a cutting seton made of horsehair, the surgical rationale has always been the same. Nowadays the main surgical options are fistulotomy, fistulectomy and loose or cutting seton insertion. Seton insertion is often performed only to prevent further abscess formation, and the laying-open remains the efficacious surgical treatment of the fistula-in-ano. On the other hand the best choice depends on the anatomical characteristics of the fistula, and particularly if the fistulous tract crosses the external sphincter. In this case the surgical dissection could lead to severe sphincter damage with sequent fecal incontinence if the sphincter injury is too extensive. From this point of view, in order to prevent such a sphincter damage causing fecal incontinence, the most fundamental issue is to quantify the remaining functional sphincter. Only when at least 1-2 cm of functional sphincter are saved from surgical dissection the laying-open can be performed without affecting fecal continence. Otherwise the seton drainage with secondary fistulotomy, staged fistulotomy or sliding flap advancement has to be considered in order to reduce the risk of postoperative incontinence. Anyway there are still many patients suffering from postoperative permanent disturbance in anal continence, which is mostly represented by loss of flatus control and soiling, and only seldom by severe fecal incontinence. Nevertheless, a temporary early postoperative incontinence, which improves within 2-3 weeks, is a frequent complication after surgery of any fistulous tract dissecting the sphincter [1-11] (Table 1). Another postoperative complication after surgical treatment of an anal fistula is the recurrence (0-9%) [1-11] (Table 1). Basically it depends on an ineffective surgical treatment but also on the fistula etiology. For these reasons newer sphincter-saving approaches have been applied in the treatment of perianal fistula in order to avoid the risk of fecal incontinence, particularly in patients with high risk. Among these approaches the fibrin glue application is standing out because it is a simple and repeatable technique, whose success rate is improved by repeated injections, and does not interfere or compromise subsequent surgical options. Moreover the prolonged discomfort associated with wound dressing after surgery may be avoided. The first series studies about the fibrin glue treatment of anal fistulas were published by Abel and Hjortrup in the early 90' years [12,13]. Respectively they reported 60% (in 10 patients) and 52% (in 15 patients) healing rates and they both stressed out the importance of thorough curettage to remove all granulation tissue and debris, as well as a wide antibiotic administration. More recent studies do not report the same successful use of this technique, showing low healing rates [14-17]. The aim of this review is to assess the cure and complication rate of fibrin glue application compared to surgery alone for the treatment of perianal fistula.

**Table 1:** Results and complications after surgical treatment of fistula-in-ano.

*Study*	*Patients (n)*	*Recurrence (%)*	*Disturbance in anal continence (%)*
Aguilar [1]	189	0.01	0
Bennett [2]	108	2	36
Hill [3]	626	1	4
Koscinski [4]	55	6	0
Khubchandani [5]	137	5.8	-
Lilius [6]	150	5.5	13.5
Marks and Ritchie [7]	793	-	17-31
Mazier[8]	1000	3.9	0.01
McElwain [9]	1000	3.6	7-3.2
Parks and Stitz [10]	400	9	-
Pearl [11]	1732	1.8	-

## Methods of meta-analysis

### Search methods for identification of studies

We planned to search for published randomized and controlled clinical trials with no language restrictions, by using the following electronic databases: Cochrane Central Register of Controlled Trials (CENTRAL), MEDLINE (1950 onwards) and EMBASE (1980 onwards). The literature searches were carried out using medical subject headings (MeSH) and free-text word: "rectal fistula"; "anal fistula"; "fibrin adhesive"; "fibrin glue"; "fibrin sealant", "anal fistula plug", "collagen fistula plug". We also checked the reference lists of all the studies identified through the above mentioned methods. The abstracts presented to the following international scientific societies were hand searched: American College of Surgeons (2000 to 2007), American Society of Colon-Rectal Surgeons (1991 to 2007) and Società Italiana di Chirurgia (1985 to 2007), Società Italiana di Chirurgia Colon-Rettale (2006 to 2007), The Courrier de colo-proctologie.

### Data Extraction

Two authors (RC, EF) assessed titles or abstracts of all the studies identified by the initial search and excluded irrelevant studies. Full text articles of potentially relevant studies and any studies with unclear methodology were obtained. The two authors assessed all these studies as to whether they met the inclusion criteria for this review, and they evaluated the method of randomization and the adequacy of allocation concealment. Disagreements on inclusion of the studies were solved by discussion and, if necessary, by involving an independent third author (FS). The following information were independently extracted by the two investigators (RC, EF) for each included study: the primary outcome, the number of event of interest, the population included, and information on quality measure including allocation concealment, blinding of outcome evaluators, intention to treat and balance of prognostic factors.

### Inclusion Criteria

To be included in the analysis, studies had to compare conventional surgical treatment versus fibrin glue treatment in patients with anal fistulas.

### Exclusion Criteria

Studies were excluded from the analysis if: 1) the outcomes of interest were not reported for the two techniques, 2) it was impossible to extrapolate or calculate the necessary data from the published results, 3) there was considerable overlap between authors, centres, or patient cohorts evaluated in the published literature. Moreover the studies in which fibrin glue was used in the flap repair of anal fistulas were also excluded from this review.

### Outcomes of Interest

The following outcomes were used to compare the: 1. Clinical healing of fistula 2. Anal incontinence.

### Methodological quality

EF and RC recorded whether the Authors of the trials used a sample size calculation, or they performed their analysis using an intention-to-treat method.

### Assessment of the methodological quality of the studies

The review authors followed the instructions given in the Cochrane Handbook for Systematic Reviews of Interventions.

### Measures of treatment effect

Dichotomous data were analyzed for relative risk ratio (RR), odds ratio (OR), and the absolute results were measured with the risk differences retrieved to calculate the odds ratio (OR) and 95% confidence intervals (CI) were calculated. The Mantel-Haenszel method was used for the meta-analysis. Results were presented on a forest plot graph.

### Assessment of heterogeneity

Chi-squared test was used for heterogeneity assessment. If different trials used different scales, the results were standardized and then combined (i.e. standardized mean difference).

### Statistical Analysis

We used Review Manager 5 to conduct the review.

## Results

### Eligible Studies

There are currently two RCTs on this issue comparing conventional surgical treatment versus fibrin glue treatment in patients with anal fistulas [18,19] (Table 2). The assessment of quality of the studies was evaluated by two assessors (RC and FS) by considering: presence of detailed criteria for assignment of patients to the surgical or fibrin glue treatment group; absence of any difference between the two groups assessing the comparability on the basis of study design or analysis of group differences; adequacy of patients' follow-up (> 6 months). All recurrences after the fibrin glue treatment appear within 3 months and only occasionally they are detectable after 6 months [20].

**Table 2:** Characteristics of the studies considered.

*Study*	*Patients*	*Treatment*	*Outcomes*
Greco [18]	77 trans sphincteric anal fistulas	32 surgical treatments (cutting seton) vs. 45 fibrin glue	Clinical healing
Lindsey [19]	13 simple fistulas (low fistulas) and 29 complex fistulas	23 surgical treatments (fistulotomy) vs. 19 fibrin glue	Clinical healing

### Results from analysis

**Figure 1:**
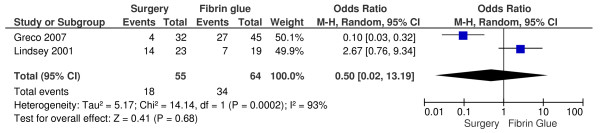
**Healing: conventional surgical treatment versus fibrin glue treatment in patients with anal fistulas**.

**Figure 2:**
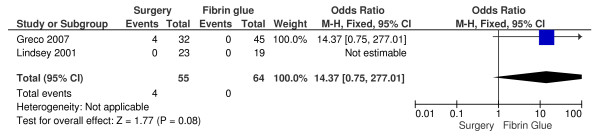
**Incontinence: conventional surgical treatment versus fibrin glue treatment in patients with anal fistulas**.

Even if it is not statistically relevant, the healing rate is higher in those patients who underwent conventional surgical treatment (odds ratio OR, 0.50; 95 percent confidence interval CI, 0.02-13.19; P = 0,68) (Figure 1). In this analysis there is a significant heterogeneity (chi-square = 14.14 - I^2 ^= 93%), and therefore for the OR calculation we used the M-H Random test instead of the fixed one. Despite of the lower healing rates in the fibrin glue treatment group, no anal incontinence is noticed (odds ratio OR, 14.37; 95 percent confidence interval CI, 0.75-277.01; P = 0,08) (Figure 2). Also this result is not statistically relevant.

## Subgroup analysis

We performed two subgroup analyses assessing other sphincter saving approaches for the treatment of fistulas-in-ano in patients with high risk of postoperative disturbance in anal continence:

- Fibrin glue in combination with intra-adhesive antibiotics vs. fibrin glue alone.

- Anal fistula plug vs. fibrin glue.

## Fibrin glue in combination with intra-adhesive antibiotics vs. fibrin glue alone

### Inclusion Criteria of subgroup analysis

To be included in this subgroup analysis the studies had to compare the fibrin glue application in combination with intra-adhesive antibiotics treatment vs. a simple fibrin glue treatment of fistulas-in ano.

### Eligible Studies for subgroup analysis

Using the key words listed above, we identified 253 abstracts. The examination of all the abstracts and their references on the basis of the inclusion criteria of this subgroup analysis, only gave us one valid study to be analyzed [21].

### Results from subgroup analysis

**Figure 3:**
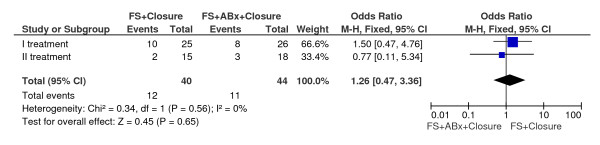
**Fibrin glue in combination with intra-adhesive antibiotics vs. fibrin glue alone**.

The analysis of Singer's study did not show differences in healing between treatment with fibrin glue in combination with intra-adhesive antibiotics and fibrin glue alone (odds ratio OR, 1.26; 95 percent confidence interval CI, 0.47-3.36; P = 0,65) [21] (Figure 3).

## Anal fistula plug vs. fibrin glue

### Inclusion Criteria of subgroup analysis

To be included in this subgroup analysis the studies had to compare anal fistula plug treatment versus fibrin glue treatment of fistulas-in ano.

### Eligible Studies for subgroup analysis

The examination of the 253 abstracts and their references previously identified on the basis of the inclusion criteria of this subgroup analysis, allowed us to get only 1 valid study to be analyzed [22].

### Results from subgroup analysis

**Figure 4:**
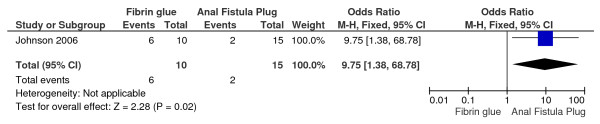
**Anal fistula plug vs. fibrin glue**.

In Ky's study the healing rate is statistically significant in the patients who underwent the anal fistula plug treatment of fistulas-in ano (odds ratio OR, 9.75; 95 percent confidence interval CI, 1.38-68.78; P = 0,02) [22] (Figure 4).

## Discussion

Fibrin glue treatment of anal fistulas is simple, safe, and painless and the injections can be repeated to increase the healing rate without preventing from other eventual following surgical procedures. For these reasons in the past decade this technique became increasingly popular, but today many doubts about fibrin glue procedure still remain because of its poor long-term results [23-25].

Our statistical analysis confirms the poor long-term results in the patients who underwent fibrin glue treatment rather than surgery, considering both healing and not-healing after an adequate 6-month follow-up, regardless of the fact that the not-healing rate would be a recurrence or not. We did not calculate any recurrence rate after fibrin glue treatment, as we could not find satisfactory data in literature about accurate recurrence rate in the two included studies. Another important point to consider about fistula healing is that the closure of the external skin wound does not always mean complete healing. Buchanan et al. reported a prospective clinical trial with 22 patients presenting the possibility to establish a complete healing of idiopathic complex anorectal fistulas after fibrin glue treatment. The Authors evaluated the fistula tract healing on the basis of the clinical examination and magnetic resonance imaging (STIR sequence MRI long-term can detect deep persistence). Despite of the skin healing in 77% of the patients at 14 days after treatment, 3 cases (14%) were clinically healed at 16 months and only 2 cases (9%) were radiologically healed at 16 months [25]. In Table 3 we analyzed the different fistulas etiologies in order to estimate the prognostic value of the etiology in the fibrin glue treatment of the perianal fistula [12,15,16,19,20,26-28]. Even though most of the studies in literature include fistulas of mixed etiologies, cryptoglandular fistulas are the major group, whereas no-cryptoglandular fistulas represent only a small amount. The fistula healing rate varies widely (0-100%) in Crohn disease patients. In HIV-associated and rectovaginal fistulas the healing rate appears to be poor, although the number of patients considered is too low to obtain certain results. The number of ileal-pouch anal anastomosis fistula patients treated with fibrin glue is very low as well, but in these patients the results seem to be good. On the other hand it was possible to estimate the prognostic value of the anatomical characteristics of the fistula, particularly comparing simple or complex fistulas (Table 4) [12,15,16,19,20,23-28]. Our analysis shows that simple fistulas have a better healing rate than complex fistulas.

**Table 3:** Healing rates after treatment with fibrin glue for fistula in ano.

	Cryptoglandular	Crohn's disease	HIV	Rectovaginal	Ileal-pouch anal anastomosis
Abel [12]	2/3 (66%)	1/3 (33%)			
Sentovich [15]	25/36 (70%)	4/5 (80%)			
Loungnarath [16]	5/22 (23%)	4/13 (31%)		1/3 (33%)	3/4 (75%)
Lindsey [19]	10/17 (59%)	2/2 (100%)			
Cintron [20]	44/68 (65%)	2/6 (33%)	1/2 (50%)	1/3 (33%)	
Venkatesh [26]	12/15 (80%)	0/6 (0%)			
Patrij [27]	51/69 (74%)				
Zmora [28]	2/10 (20%)	2/5			2/4 (50%)

Total	154/240 (64%)	13/37 (35%)	1/2 (50%)	2/6 (33%)	5/8 (63%)

**Table 4:** Healing rates after treatment with fibrin glue in complex and simplex fistula

	Complex only		Simple and complex	
	Healed	Not healed	Healed	Not healed
Abel [12]	6	4		
Sentovich [15]			33	15
Loungnarath [16]	12	27		
Lindsey [19]	9	13		
Cintron [20]			48	31
Zmora [23]	32	28		
Tinay [24]			19	25
Buchanan [25]	3	19		
Venkatesh [26]	18	12		
Patrij [27]			51	18
Zmora [28]	8	16		

Total	88 (42.5%)	119 (57.5%)	151 (63%)	89 (37%)

Recent articles report encouraging results, which will need further studies to be confirmed, in the repair of anal fistulas by using Surgisis AFP (Anal Fistula Plug: Cook Medical Incorporated, Bloomington, IN), a bioabsorbable plug derived from porcine small submucosa (SIS) [29,30]. The plug is placed into the fistula tract and sutured to the internal opening. SIS promotes tissue remodelling while being slowly incorporated into the body during a 3 to 6-month period. Surgisis AFP long-term closure rate is significantly higher in patients with simple fistulas than complex ones and with non-Crohn disease versus Crohn disease [22]. The major complication of Anal Fistula Plug is a severe perianal sepsis (14.7% Ky 2008 - 29% Lawes 2008) requiring surgical drainage and removal of the plug [22,30].

## Conclusion

Nowadays the laying-open, seton insertion, staged fistulotomy and sliding flap advancement are still the main pillar of perianal fistula surgery, while fibrin glue alone or in combination with intra-adhesive antibiotics and the anal fistula plug would rather be used in the patients with high risk of postoperative disturbance of anal continence. In literature we found only two randomized controlled trials comparing conventional surgical management versus fibrin glue treatment in patients with anal fistulas. Although from our statistical analysis we could not find any statistically significant result, the healing rate is higher in the patients who underwent the conventional surgical treatment (P = 0,68), and the anal incontinence rate is very low in the fibrin glue treatment group (P = 0,08). Anyway these limited data do not support the use of fibrin glue. Besides, in our subgroup analysis the use of fibrin glue in combination with intra-adhesive antibiotics does not improve the healing rate (P = 0.65), whereas the anal fistula plug treatment compared to the fibrin glue treatment shows good results (P = 0,02), although the poor number of patients treated does not lead to any significant conclusion. Our systematic review underlines the need of new RCTs upon this issue.

## Competing interests

The Authors state that none of the authors involved in the manuscript preparation has any conflicts of interest towards the manuscript itself, neither financial nor moral conflicts. Besides none of the authors received support in the form of grants, equipment, and/or pharmaceutical items.

## Authors' contributions

All authors contributed equally to this work, read and approved the final manuscript.
